# Expression of a rice chitinase gene in transgenic banana (‘Gros Michel’, AAA genome group) confers resistance to black leaf streak disease

**DOI:** 10.1007/s11248-012-9631-1

**Published:** 2012-07-13

**Authors:** Gabriella Kovács, László Sági, Géraldine Jacon, Geofrey Arinaitwe, Jean-Pierre Busogoro, Els Thiry, Hannelore Strosse, Rony Swennen, Serge Remy

**Affiliations:** 1 Laboratory of Tropical Crop Improvement, Department of Biosystems, Faculty of Bioscience Engineering, KU Leuven, Kasteelpark Arenberg 13, bus 2455, 3001 Leuven, Belgium; 2 Plant Cell Biology Department, Centre for Agricultural Research, Hungarian Academy of Sciences, 2462, Martonvásár, Brunszvik u. 2, Hungary; 3 Interface Entreprises, Université de Liège, Avenue Pré-Aily 4, 4031 Liège, Belgium; 4 National Agricultural Research Laboratories, Kawanda, P.O. Box 7065, Kampala, Uganda; 5 Belgian Technical Cooperation (BTC-CTB), 41 Rue Député Kayuku, BP 6089, Kigali, Rwanda; 6 Interleuven, Intergemeentelijke Vereniging, Brouwersstraat 6, 3000 Leuven, Belgium; 7 Bioversity International, KU Leuven, Kasteelpark Arenberg 13, 3001 Leuven, Belgium

**Keywords:** Black leaf streak disease, Leaf disk assay, *Musa* spp., *Mycosphaerella fijiensis*, Transgenic banana

## Abstract

Transgenic banana (*Musa acuminata* ‘Gros Michel’) integrating either of two rice chitinase genes was generated and its resistance to Black Leaf Streak disease caused by the fungus *Mycosphaerella fijiensis* was tested using a leaf disk bioassay. PCR screening indicated the presence of the *hpt* selectable marker gene in more than 90 % of the lines tested, whereas more than three quarters of the lines contained the linked rice chitinase gene resulting in a co-transformation frequency of at least 71.4 %. Further, a unique stable integration of the transgenes in each line revealed some false negative PCR results and the expected co-transformation frequency of 100 %. The transgene insert number per line ranged from 1 to 5 and single transgene insert lines (25 % of all) were identified. Considerable delay in disease development (up to 63 days post-incoculation) over a monitoring period of 108 days occurred in nine lines with extracellularly targeted chitinase out of 17 transgenic lines tested and their necrotic leaf area decreased by 73–94 % compared to the untransformed susceptible control line. Finally, correlation between symptom development and rice chitinase expression was confirmed in two lines by Western analysis. The potential of rice chitinase genes to enhance resistance against *M. fijiensis* in banana was demonstrated as well as the usefulness of the leaf disk bioassay for early disease screening in transgenic banana lines.

**Electronic supplementary material:**

The online version of this article (doi:10.1007/s11248-012-9631-1) contains supplementary material, which is available to authorized users.

## Introduction

Banana is the main fruit in international trade in terms of volume and the fourth most important staple crop with a world production of 133 million tons in 2009 (FAOSTAT 2011a). Hence, the importance of banana (including plantain) production in economy and sustenance of developing countries in the tropical and subtropical regions is beyond dispute. More than 60 % of total banana production is concentrated in a few countries like India, China, the Philippines, Brazil and Ecuador (UNCTAD), although it is produced in about 120 countries worldwide. Nonetheless, a mere 20 % of global banana production enters international trade (UNCTAD) representing a total value of US$ 8.3 billion in 2009 (FAOSTAT 2011b). The remaining yield is consumed by local population in Asia, America and Africa as a major staple food. Uganda is one of the largest producers in Africa with more than 10 million metric tons harvested over 1.8 million ha in 2010 (CountrySTAT 2011). In addition, the country has a longstanding record of the highest banana consumption rate in the world with presently a daily average of 0.6 kg per capita. Over the last decade, banana yields in Uganda have stagnated around 6 t ha^−1^ (CountrySTAT 2011) while the population has increased by 35 % in the same period. At least half of the population directly depends on banana as staple food and/or as source of daily income. Thus, there is a real and urgent need for increased productivity in the banana sector for this region. Though East African highland cultivars are the primary bananas, which are used to prepare the principle staple food ‘matooke’, ‘Gros Michel’ (local name: ‘Bogoya’) is still a popular dessert banana. Moreover, ‘Gros Michel’ was once the most important commercial dessert cultivar until the 1960s when it was replaced by Cavendish-type bananas due to the outbreak of Panama wilt caused by *Fusarium oxysporum* f.sp. *cubense* Race 1.

In addition to pests, bacterial and viral diseases the production of banana is severely threatened worldwide by a leaf attacking hemibiotroph fungal pathogen belonging to the *Ascomycetes* division: *Mycosphaerella* *fijiensis* Morelet, causing the Black Leaf Streak Disease (BLSD), also known as Black Sigatoka disease. During the last few decades, widespread global distribution of *M. fijiensis* has been reported including even subtropical areas as it is able to infect almost all edible banana types because of lack of resistance (Jones 2009; Ploetz 2001). Yield losses attributed to BLSD gradually grew to more than 50 % (Marciel Cordeiro and Pires de Matos 2003; Ploetz 2001), while chemical control of the disease in commercial plantations increased production costs up to $1,800 per ha [25–30 % of the total (Marín et al. 2003)], which is not affordable for small growers in developing countries. Fungicide spraying has a major impact not only on the environment and human health (Castillo et al. 2000; Wesseling et al. 2005) but on cultivation as well due to the development of fungus resistance to the active ingredients (Cañas-Gutiérrez et al. 2006). As a result, a commercial plantation may nowadays require more than 60 sprays annually. The use of resistant, agronomically acceptable cultivars in commercial and small farm production would be the only practical approach to sustainable control of BLSD (Jones 2009; Ploetz 2001).

Classical breeding for disease resistance is extremely difficult and time consuming in banana due to a long generation cycle (up to 2 years), polyploidy, male sterility and highly reduced female fertility of local cultivars (Swennen and Vuylsteke 1993). With the development of embryogenic cell suspension cultures (Côte et al. 1996) and high-throughput transformation methods using *Agrobacterium* (Pérez Hernández et al. 2006) genetic modification has become a promising additional tool for banana improvement. Consequently, introducing potential resistance genes against various banana diseases has been attempted and developing a gene transfer-based technology that provides biotic stress resistance to banana cultivars without changing their genetic makeup is of prime importance (Chakrabarti et al. 2003; Remy et al. 1998a; Vishnevetsky et al. 2011).

The most widely employed transgenic approach to enhance resistance against fungal diseases has been based on the over-expression of pathogenesis related (PR) proteins, among which plant chitinases have been the most extensively studied and applied since 1991 (Broglie et al. 1991). Chitinases, the first identified group of PR proteins, are abundant proteins known to exert antifungal activities by degrading chitin, a major component of the cell wall of fungal phyla *Basidiomycota* and *Ascomycota* (including their asexually reproducing members that were previously ranked in a the separate phylum Deuteromycota or Deuteromyces) as well as *Zygomycota,* which is absent in plants (Neuhaus 1999; Punja 2004). Among the numerous plant chitinase genes used in transformation experiments (for overview, see Table 7.1 in Punja 2004) rice chitinases have been the most studied. Resistance to a range of pathogenic fungi by rice chitinase transgenes has been demonstrated in economically important plants such as strawberry (Asao et al. 1997), rose (Marchant et al. 1998), chrysanthemum (Takatsu et al. 1999), rice (Datta et al. 2001; Lin et al. 1995; Nishizawa et al. 1999), grapevine (Yamamoto et al. 2000), sorghum (Krishnaveni et al. 2001), cucumber (Kishimoto et al. 2002) and Italian ryegrass (Takahashi et al. 2005).

When transgenic plant material is generated for disease resistance it is essential to confirm the resistant phenotype prior to expensive field testing. Leaf disk bioassays have been extensively applied to assess the level of resistance against different fungal pathogens under controlled conditions. This tool has proven effective to screen for natural resistance in diverse plant/pathogen (biotroph, hemibiotroph, necrotroph) systems such as coffee/*Hemileia vastatrix* (Eskes 1982), potato/*Alternaria solani* (Bussey and Stevenson 1991), maize/*Cochliobolus carbonum* (Meeley et al. 1992), melon/*Sphaerotheca fuliginea* (Cohen 1993), cauliflower, broccoli/*Peronospora parasitica* (Agnola et al. 2003) and banana/*Mycosphaerella fijiensis* (Abadie et al. 2009). In the case of banana, symptoms of *M. fijiensis* infection appear in the field after 2–4 weeks of incubation and proceed for 1–2 more months. Therefore, maintaining viability of excised leaf disks for at least 2–3 months is a must for the development of a useful bioassay.

The aim of this study was twofold: firstly,we developed stable transformation of ‘Gros Michel’ via highly embryogenic and regenerable cell suspension cultures; and secondly, we explored enhanced BLSD resistance in banana via genetic transformation with heterologous genes. Using our optimized *Agrobacterium*-mediated transformation system (Pérez Hernández et al. 2006), we report here for the first time on the production of transgenic ‘Gros Michel’ plants, which carry either the *rcc2* or *rcg3* class-I rice chitinase gene (Nishizawa et al. 1999). After molecular characterization, transgenic lines were tested for resistance by an optimized leaf disk assay (LDA). Lines with significantly less necrotic leaf area than the untransformed control for up to 108 days after *M. fijiensis* inoculation were identified and the expression of the chitinase (RCG3) protein was immunologically confirmed.

## Materials and methods

### Plant material

Embryogenic cell suspensions (ECS) of the dessert banana cultivar ‘Gros Michel’ were maintained in liquid ZZ medium containing 5 μM 2,4-d and 1 μM zeatin (Dhed’a et al. 1991). The suspension was initiated from male buds (Côte et al. 1996) at CIRAD (Montpellier, France). Cells were maintained on a rotary shaker (70 rpm) at 26 ± 2 °C under continuous light of 50 μE m^−2^ s^−1^ and subcultured at an interval of 2 weeks.

### Binary vectors, *Agrobacterium* strains, in vitro culture and transformation

The binary vectors pBI333-EN4-RCC2 and pBI333-EN4-RCG3 (Nishizawa et al. 1999) contain the hygromycin phosphotransferase (*hpt*) gene driven by the CaMV35S promoter and one of two rice chitinase (*rcc2* or *rcg3*) genes fused to an enhanced CaMV35S promoter. The two vectors were transferred into *Agrobacterium tumefaciens* strain EHA105 (Hood et al. 1993) as previous experiments had shown this strain to infect ECS cells of a broad range of banana cultivars (Pérez Hernández et al. 1999).

Bacteria were cultured at 28 °C for 48 h on solid yeast-mannitol medium (0.4 g L^−1^ yeast extract, 10 g L^−1^ mannitol, 0.5 g L^−1^ K_2_HPO_4_·3H_2_O, 0.2 g L^−1^ MgSO_4_·7H_2_O, 0.1 g L^−1^ NaCl, pH 7.0) containing 50 mg L^−1^ kanamycin. Single colonies were picked and shaken in selective liquid yeast-peptone medium (10 g L^−1^ yeast extract, 10 g L^−1^ peptone, 5 g L^−1^ NaCl) at 28 °C and 210 rpm for 30 h. *Agrobacterium*-mediated transformation of banana ECS cultures followed by selection and regeneration of transgenic lines was performed as reported previously (Pérez Hernández et al. 2006).

### Total DNA isolation

Total banana DNA was isolated by a combination of two described protocols (Aljanabi and Martinez 1997; Dellaporta et al. 1983). Fresh leaf samples were collected from the greenhouse, homogenized in liquid nitrogen with mortar and pestle, and processed in the extraction buffer [100 mM Tris–HCl, pH 8.0, 50 mM EDTA, 500 mM NaCl, 10 mM β-mercaptoethanol, 2 % (w/v) polyvinyl pyrrolidone (MW 10,000)]. The integrity of the dissolved DNA was observed after 0.8 % (w/v) agarose gel electrophoresis, and the concentration and quality was spectrophotometrically determined.

### Polymerase chain reaction

PCR was performed in 0.2 mL microfuge tubes in a Mastercycler Gradient™ cycler (Eppendorf, Hamburg, Germany). The final volume of 20 μL was made up of 50 ng of plant DNA template (in 2 μL Milli-Q water) and 18 μL of master mix, which consisted of 1× MgCl_2_ containing PCR buffer (QIAGEN, Hilden, Germany), 0.2 mM dNTPs, the gene-specific primers (Online Resource 1) at a final concentration of 0.5 μM and 0.025 U μL^−1^ *Taq* DNA polymerase (QIAGEN). Reactions were programmed to an initial denaturation for 2 min at 94 °C and then to 35 cycles as follows: 1 min at 94 °C, 30 s at 56 °C and 1 min at 72 °C. The final elongation step was prolonged to 7 min at 72 °C. Positive controls (binary vector DNA) as well as two negative controls (water and untransformed plant DNA) were included in each experiment.

### Southern blot hybridization

For Southern analysis, enzymatic digestion of 10 μg plant and plasmid DNA (10 pg) with *Hin*dIII that cuts once in the T-DNA was followed by electrophoresis on 0.8 % (w/v) agarose gel and transfer of separated fragments to a positively charged nylon membrane (Roche, Vilvoorde, Belgium) by downward capillary blotting. Probes were labelled with digoxigenin-dUTP (Roche) by PCR. To eliminate unspecific hybridization signals, more stringent membrane washes were performed at a higher temperature (70 °C) and lower salt concentration: 1× SSC and 0.1 % (w/v) SDS, twice for 30 min, followed by 0.05× SSC and 0.1 % SDS, twice for 30 min. Signal detection with the CSPD^®^ chemiluminescent substrate was done according to the manufacturer’s instructions (Roche); signals were captured with a cooled CCD camera (Versarray™ 512 B LN, Roper Scientific, Vianen, The Netherlands).

### Leaf disk assay

Inoculum of *Mycosphaerella fijiensis* (Costa Rica strain R1 ‘Yangambi Km5’ isolate) was produced by first plating several plugs carrying a highly sporulating *M. fijiensis* culture stored at −80 °C on sterile V8 medium (100 mL L^−1^ V8 juice, 0.2 g L^−1^ CaCO_3_ and 20 g L^−1^ agar) supplemented with 1 mL L^−1^ of filter-sterilized antibiotic cocktail of 120 mg L^−1^ penicillin and 200 mg L^−1^ streptomycin. The cultures were incubated at 24 °C under a 12-h photoperiod (light intensity of 45 μE m^−2^ s^−1^, above the Petri plates) for 10 ± 2 days until appearance of melanin-rich colonies. Twenty colonies were lifted with a scalpel and transferred to a test tube containing 10 mL distilled water. After rigorous vortexing, 1 mL suspension containing mycelium fragments and conidiospores was plated on fresh V8 medium and incubated at 24 °C under a 12-h photoperiod until fungal growth covered the whole plate. For large-scale inoculum preparation, the sporulating cultures were flooded with sterile distilled water (4–5 mL per Petri dish) and gently scraped using the backside of a scalpel blade to harvest the conidiospores. The concentration of conidia was determined in a haemocytometer and adjusted with sterile distilled water to 2 × 10^4^ conidiospores mL^−1^. Ready suspensions were loaded into a regular flower sprayer of 1 L.

The youngest fully expanded leaf was harvested from 9-month old ‘Gros Michel’ plants (transgenic and control) grown under greenhouse conditions and washed with distilled water. Twelve leaf squares of 5 × 5 cm were excised per leaf and surface sterilized in 1 % (w/v) NaOCl solution for 90 s followed by five-six rinses with distilled water. The leaf disks were then placed with the upper (adaxial) side down on 0.4 % (w/v) water agar supplemented with 8 mg L^−1^ filter-sterilized gibberellic acid (GA_3_) (Twizeyimana et al. 2007a) in plastic Petri dishes. Ten out of 12 leaf disks per line were inoculated by spraying the conidial suspension on the abaxial leaf surface until saturated with small droplets. The remaining two leaf disks were not sprayed and served as non-infected internal control to distinguish lesions caused by *M. fijiensis* from those induced by wounding or other stress. Petri dishes were sealed with cling film and incubated at 24 °C under 12-h photoperiod (at 25 μE m^−2^ s^−1^) until the final observation of disease symptoms. As a resistant control, leaf disks from the wild diploid ‘Calcutta4’ (International Transit Centre accession no. ITC0249) were also prepared as described above.

Lesion development was monitored starting from the appearance of the first visible symptoms on the most susceptible transgenic lines (39 days post inoculation, dpi). Photos were taken at 10 to 14-day intervals (until 108 dpi) to record the progress of the necrotic lesions. Images were analyzed with the ASSESS software (Lamari 2009), and the relative infected leaf area, including the chlorotic halo, was expressed in percentage of the total leaf disk area.

### Western analysis

Leaf tissues were collected from the youngest fully expanded leaf of 6-month old greenhouse plants (an in vitro propagated progeny of transgenic and control plants used in the above-mentioned LDA) and washed with distilled water. Four leaf squares of 5 × 5 cm excised per leaf were surface sterilized and incubated on 0.4 % (w/v) water agar supplemented with 8 mg L^−1^ filter-sterilized GA_3_ in plastic Petri dishes (as described above) under non-infected conditions. Leaf disks were collected at 1, 29, 55 and 78 days post-incubation and stored at −80 °C until analysis. Total protein was extracted as described (Wang et al. 2006) after grinding in liquid nitrogen with a mortar and pestle. Protein concentration in the soluble protein extract was determined with the 2-D Quant kit (GE Healthcare, Diegem, Belgium). Proteins (30 μg) were separated on NuPage 12 % Bis–Tris gels (Invitrogen, Merelbeke, Belgium) and transferred to Hybond™-ECL membrane (GE Healthcare). The polyclonal antibody to detect the RCC2 and the RCG3 protein was kindly provided by Dr. Y. Nishizawa. The ECL chemi-luminescent detection system (GE Healthcare) was used to detect the proteins and signals were captured with a cooled CCD camera (Versarray™ 512 B LN, Roper Scientific).

### Statistical analysis

Infected leaf area data were analyzed by Kruskal–Wallis test, a non-parametric alternative of ANOVA, because Levene’s test for homogeneity of variances was not significant after transformation of the data. Data were analyzed at every time point and each transgenic line was compared to the susceptible ‘Gros Michel’ control line.

## Results

### Production of transgenic plants

Transformation of ‘Gros Michel’ ECS with two rice chitinase genes, *rcc2* or *rcg3*, resulted in hundreds of putative transgenic colonies on hygromycin containing medium. For each construct 120 colonies were harvested, of which 26 (21.6 %) and 39 (32.5 %) independent plants regenerated on selective medium, respectively. During maintenance and multiplication some of the plants got contaminated and lost, decreasing the final number of transformed lines to three for *rcc2* and 21 for *rcg3*.

### Molecular characterization of transgenic plants

The presence of the selectable marker gene (*hpt*) and each of the chitinase genes was analyzed in all putative transformed ‘Gros Michel’ lines (Table 1). Fifteen out of the 21 *rcg3* lines proved positive for the two transgenes, whereas four lines were positive for only the *hpt* transgene, one for the *rcg3* transgene only and one line remained negative for both transgenes. Hence, transformation frequencies of 90.5 and 76.2 % were achieved for the *hpt* and *rcg3* transgene, respectively. Although a co-transformation frequency of 100 % could theoretically be expected for these linked genes, it reached only 71.4 % due to false negative results (see Table 2). Further, all three *rcc2* lines proved to be positive for both transgenes and thus, the transformation as well as the co-transformation frequencies were 100 % in these ‘Gros Michel’ transformants.

**Table 1:** Transformation frequencies of the transgenes in putative transformed ‘Gros Michel’ lines as determined by PCR

Transgene^a^	No. of tested lines	(Co-)transformation frequency (%)^b^		
		*rcg3* transformants	*rcc2* transformants	Co-transformation with *hpt*
*hpt*	24	90.5	100	NA^c^
*rcg3*	21	76.2	NA^c^	71.4
*rcc2*	3	NA^c^	100	100

^a^In the *rcg3* and *rcc2* lines the *hpt* selectable marker gene was linked with the respective rice chitinase transgene

^b^Calculated as the ratio between the number of independent transformants containing (both) the transgene(s) and the number of independent transformants analyzed

^c^Not applicable

**Table 2:** Screening by PCR for the presence of transgenes and their estimated number of insertions as determined by Southern hybridization in 24 transgenic ‘Gros Michel’ lines

Transgenic line	PCR analysis		Southern hybridization (estimated number of transgene inserts)	
	*hpt*	*rcg3*	*hpt*	*rcg3*
GM.RCG3.01^a^	+	−	1	2
GM.RCG3.04	+	+	2	2
GM.RCG3.06	+	+	1	2
GM.RCG3.07 GM.RCG3.09	+ +	+ +	4 1	4 2
GM.RCG3.10	+	+	2	2
GM.RCG3.11 GM.RCG3.15^a^	+ +	+ −	1 2	1 2
GM.RCG3.17 GM.RCG3.20	+ +	+ +	5 2	4 3
GM.RCG3.21	+	+	2	1
GM.RCG3.24	+	+	2	2
GM.RCG3.27^a^ GM.RCG3.28^a^ GM.RCG3.29	− + +	− − +	2 1 5	1 1 4
GM.RCG3.31	+	+	3	4
GM.RCG3.32^a^	+	−	1	2
GM.RCG3.34^a^ GM.RCG3.35	− +	+ +	3 5	4 5
GM.RCG3.38	+	+	2	3
GM.RCG3.39	+	+	2	1
GM control	−	−	−	−
Average			2.3	2.5
	*hpt*	*rcc2*	*hpt*	*rcc2*
GM.RCC2.02	+	+	2	1
GM.RCC2.10 GM.RCC2.14	+ +	+ +	1 4	2 4
GM control	−	−	−	−
Average			2.3	2.3

^a^The presence and integration of the *hpt* and/or *rcg3* transgene in the genome of PCR-negative transgenic lines was confirmed by Southern hybridization

**Fig. 1:**
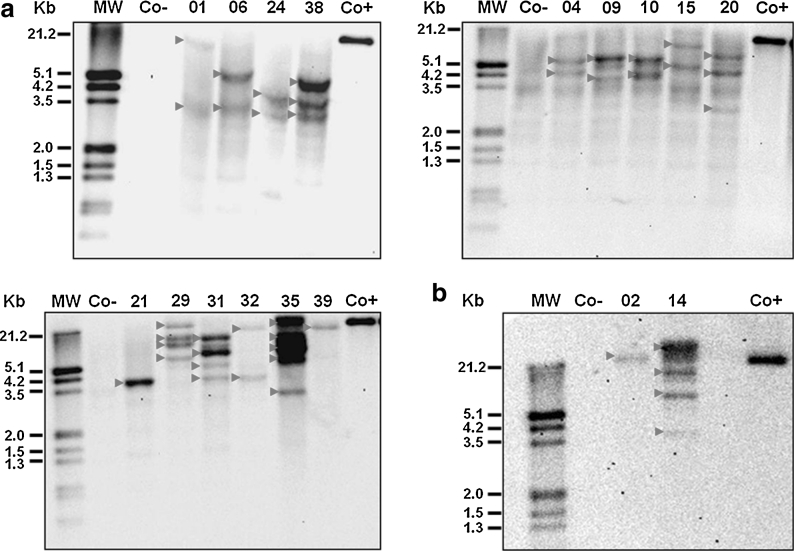
Southern blot analysis for the integration of the *rcg3* (**a**) or *rcc2* (**b**) genes in independent transgenic ‘Gros Michel’ lines. Total DNA (10 μg) was digested with *Hin*dIII and hybridized with an *rcg3* or *rcc2*-specific probe that spans part of the enhanced 35S promoter and the 5′ end of the *rcg3* or *rcc2* gene. A *red arrowhead* represents an *rcg3* or *rcc2* integration. MW: DIG-labeled molecular size marker (Roche); Co-: untransformed control plant; Co+: *Hin*dIII digested pBI333-EN4-RCG3 (**a**) or pBI333-EN4-RCC2 (**b**) plasmid controls (10 pg each); numbered lanes: independent transgenic lines **a** 01:GM.RCG3.01; 04:GM.RCG3.04; 06:GM.RCG3.06; 09:GM.RCG3.09; 10:GM.RCG3.10; 15:GM.RCG3.15; 20:GM.RCG3.20; 21:GM.RCG3.21; 24:GM.RCG3.24; 29:GM.RCG3.29; 31:GM.RCG3.31; 32:GM.RCG3.32; 35:GM.RCG3.35; 38:GM.RCG3.38; 39:GM.RCG3.39; **b** 02: GM.RCC2.02; 14: GM.RCC2.14

All 24 ‘Gros Michel’ lines (21 *rcg3* and three *rcc2*) were further characterized for stable integration of the *hpt* (results not shown), *rcg3* and *rcc2* rice chitinase genes by Southern hybridization analysis (Fig. 1; Table 2). The unique *Hin*dIII restriction site in the T-DNA allowed to estimate the number of transgene inserts for each transgenic line by the number of hybridization signals. Using an *hpt* specific probe a hybridization signal was obtained for all the tested lines demonstrating stable integration of the *hpt* selectable marker gene in their genome and corroborating the PCR results (Table 1). A line-specific integration in one to five loci was shown by the number of hybridization bands (Table 2). The average *hpt* insert number in the population was 2.3, but six (28.6 %) *rcg3* lines and one *rcc2* line contained a single *hpt* insertion. Careful selection of probes spanning part of the enhanced 35S promoter and the 5′ end of each rice chitinase gene combined with stringent washing conditions (see Sect. Materials and methods: Southern blot hybridization) minimized cross-hybridization with homologous banana chitinase genes as shown by the lack of hybridization signals in the untransformed control lines (Fig. 1, Co-lanes). Hence, the number of hybridization bands demonstrated that one to five inserts of the *rcg3* gene and one to four inserts of the *rcc2* gene were integrated in the genome (Table 2). Five (23.8 %) out of 21 *rcg3* lines and one of the three *rcc2* lines harboured a single chitinase gene insert. Stable integration of the *rcg3* gene was observed in all *rcg3* lines demonstrating false negative PCR results in five of them. As expected for linked genes, these results are in agreement with the stable integration of the *hpt* selectable marker gene in each line (Table 2). In the majority of the lines (15/24, 63 %) slightly different numbers of inserts for the two linked transgenes were found, indicating a low degree of incomplete T-DNA integration and/or rearrangements. Finally, unique integration patterns (Fig. 1) confirmed that all transgenic ‘Gros Michel’ lines were independent lines.

### Leaf disk bioassay to assess resistance to *M. fijiensis* and evaluation of disease symptoms

**Fig. 2:**
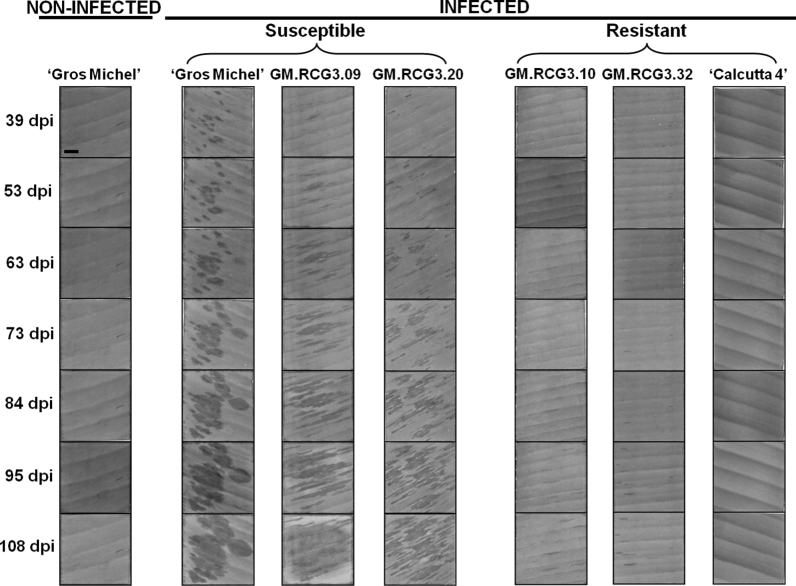
Leaf disk bioassay with *Mycosphaerella fijiensis* on 9-month old greenhouse ‘Gros Michel’ banana plants transformed with one of two rice chitinase genes. Twelve leaf disks (5 × 5 cm) were placed on 0.4 % (w/v) water agar supplemented with GA_3_ (8 mg L^−1^). The abaxial surface of ten replicates per line was sprayed with suspension of *M. fijiensis* conidiospores (2 × 10^4^ mL^−1^) and the other two served as non-inoculated internal controls. Untransformed ‘Gros Michel’ and ‘Calcutta 4’ plants were susceptible and resistant controls, respectively. Images were taken at seven time points (39, 53, 63, 73, 84, 95 and 108 days post inoculation = dpi). GM.RCG3.09 and GM.RCG3.20 were the two most susceptible transgenic lines, and GM.RCG3.10 and GM.RCG3.32 were the two most resistant ones in comparison with the untransformed ‘Gros Michel’ and ‘Calcutta4’ controls. RCG3, transgenic lines with rice chitinase *rcg3*

In total, 17 rice chitinase gene transformed ‘Gros Michel’ banana lines (15 *rcg3* and two *rcc2*) were evaluated for BLSD resistance. The first visible lesions on the susceptible control appeared 34 days after inoculation while 39 days were needed for the most susceptible transgenic lines. Thus, 39 dpi represented the starting point for the disease progress assessment. Photographs were taken at seven time points and at approximately 10–14 day intervals (39, 53, 63, 73, 84, 95 and 108 dpi) from each leaf disk to monitor the progress of the disease symptoms and measure the relative infected leaf area (see Sect. Materials and methods: Leaf disk assay). The observed lesions on the susceptible lines were morphologically similar to those of BLSD in the field while those on the ‘Gros Michel’ untransformed control were more round-shaped (Fig. 2). The protocol allowed for a long survival period of the treated material in all lines tested as detached leaf disks remained green for a 3-month period, except at the edges where a wound response occurred (Fig. 2). Hence, it was reasonable to proceed with comparative and quantitative analysis of necrosis development following *M. fijiensis* infection. Statistical analysis distinguished two consistent different groups (resistant and susceptible) among the 17 transgenic lines.

**Fig. 3:**
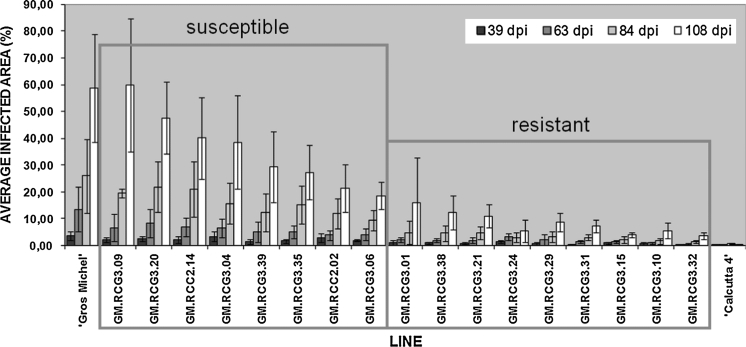
Average infected area of leaf disks 39, 63, 84, and 108 days after *Mycosphaerella fijiensis* inoculation. Experimental details under Fig. 2. Each entry is the average (±SD) of ten replicate images analyzed by ASSESS software. At each time point the data were subjected to Kruskal–Wallis test which pointed out nine transgenic lines with significantly high level of resistance against *M. fijiensis* (except for line GM.RCG3.24 at 53 dpi and 63 dpi) and eight susceptible transgenic lines (framed in *red rectangles*). RCG3, transgenic lines with rice chitinase *rcg3* and RCC2, transgenic lines with rice chitinase *rcc2;* dpi, days post inoculation

The first group consisted of nine lines that showed significantly higher resistance against *M. fijiensis* infection from 39 dpi onwards, except for line GM.RCG3.24 at 53 dpi and 63 dpi when it was ranked susceptible. By the last time point (108 dpi) the necrotic area occupied a maximum of only 16 % of the total leaf disk area in this group (Fig. 3). For all lines in this group, except GM.RCG3.29 and GM.RCG3.31, the rate of disease development on the leaf disks was strongly delayed initially (until 63 dpi) relative to the ‘Gros Michel’ control when they started to increase in size at a rate comparable or even higher than the ‘Gros Michel’ control (Online Resource 2). From the earliest time point onwards lines GM.RCG3.32 and GM.RCG3.10 were highly resistant to BLSD: the necrotic leaf area at 108 dpi reached only 3.6 and 5.6 %, respectively, which was more than ten times less than that of the susceptible control line (60 %; Fig. 3). The necrotic area at 108 dpi of GM.RCG3.15 was comparable (5.2 %) to these two highly resistant lines but slightly larger at earlier time points. Meanwhile, on some of the naturally BLSD resistant ‘Calcutta 4’ leaf disks only very small hypersensitive response-like lesions were observed at 39 dpi (data not shown). These lesions enlarged somewhat over time (Online Resource 2), but resulted in a relative infected leaf area of 0.39 % at 108 dpi. The other group encompassing eight lines with 18–60 % necrotic leaf area at 108 dpi was statistically comparable to the non-transgenic ‘Gros Michel’. Though there was no significant difference compared to the control line, these transgenic lines exhibited less necrosis, except line GM.RCG3.09. From the first time point onwards the infected leaf area of line GM.RCG3.09 was similar to the untransformed ‘Gros Michel’ control; in addition, at the end of the experiment the symptoms on line GM.RCG3.09 occupied an identical percentage of the total leaf area as the non-transgenic control (Fig. 3). Although initially (from 39 to 63 dpi) the necrotic leaf area in the transgenic lines GM.RCG3.04, GM.RCC2.02 and GM.RCG3.06 enlarged less relative to the ‘Gros Michel’ control (Online Resource 2), due to later disease development these lines still fell in the susceptible group by the end of the experiment (Fig. 3). The necrotic leaf area in the other lines of this susceptible group increased at a rate comparable to the ‘Gros Michel’ control (Online Resource 2).

### Expression of the chitinase genes in the transgenic plants

**Fig. 4:**
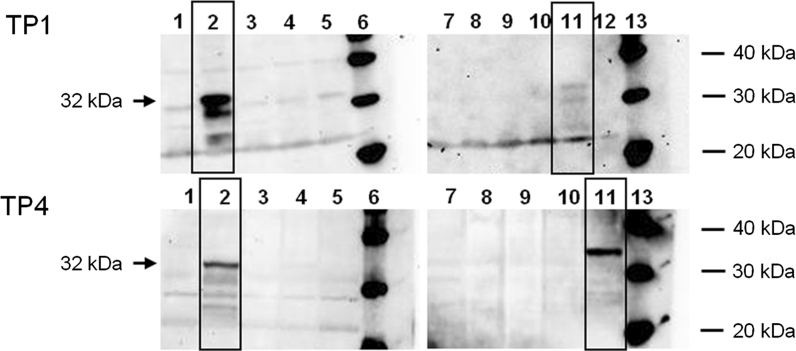
RCG3 expression in non-infected leaf disks of the most BLSD resistant transgenic ‘Gros Michel’ lines at two time points as detected by Western blot analysis. TP1: start of leaf disk assay, day 1; TP4: end of leaf disk assay, day 78; *Lane* *1* *and 7* GM untransformed control; *Lane 2* GM.RCG3.01; *Lane 3* GM.RCG3.10; *Lane 4* GM.RGC3.15; *Lane 5* GM.RCG3.21; *Lane 6 and 13* MagicMark™ Western Protein Standard (Invitrogen); *Lane 8* GM.RCG3.24; *Lane 9* GM.RCG3.29; *Lane 10* GM.RCG3.31; *Lane 11* GM.RCG3.32; *Lane 12* empty lane. *Rectangular boxes* show the positive lines

Expression of the *rcg3* chitinase gene in leaves of the nine most resistant lines (except GM.RCG3.38; Fig. 3) under non-infected conditions was analyzed by Western blotting throughout the duration of a LDA (1, 29, 55 and 78 days of incubation). A specific band of 32 kDa consistent with the molecular weight of the RCG3 protein was detected in the total protein extracts of two transformed lines (GM.RCG3.01 and GM.RCG3.32) using a polyclonal antibody raised against RCC2 (Fig. 4). Moreover, the band was detected from day 1 of the incubation onwards in these lines. A stronger signal in line GM.RCG3.01 than in line GM.RCG3.32 at 1 day of incubation turned to opposite at 78 days of incubation (Fig. 3). No signal was observed in the untransformed control and the remaining six transgenic lines. Other bands observed in the samples including the two positive lines are probably non-specific signals detected by the antibody and some of them were also present in the untransformed control. Apart from the band intensities, identical results were obtained at the four different time points (Fig. 4 and data not shown).

## Discussion

Chitinases are one of the best characterized class of PR proteins as they degrade chitin, a structural component of the fungal cell wall (Punja 2004). Despite their potential, over-expression of a plant chitinase gene for engineering resistance against BLSD has not been tested so far in banana. Here, we introduced two rice genes, each encoding a class-I chitinase, one targeted intracellularly (RCC2) and the other (RCG3) extracellularly (Nishizawa et al. 1999), into the dessert banana ‘Gros Michel’ via *Agrobacterium*-mediated transformation and challenged the transgenic plants with *M. fijiensis* in a leaf disk bioassay. By confirming the presence and stable integration of the transgenes into the banana genome, high transformation frequencies were obtained for the selectable marker gene (*hpt)* similarly to those described previously (Pérez Hernández et al. 2006; Santos et al. 2009), indicating the consistence and efficiency of the established *Agrobacterium*-mediated transformation system. Our previous experiments with particle bombardment resulted in a co-transformation frequency of at least 80 % for a linked transgene (Remy et al. 1998b) which—as expected—is lower than what obtained here with *Agrobacterium*-mediated transfer of the *rcg3* gene (100 % after Southern analysis). Chitinases are present in many plant species including banana: BLASTn searches for homologous sequences to *rcc2* in the GenBank database identified four nucleotide accessions (AJ277278, AJ277279, AF416677 and Z99966) each encoding a putative banana chitinase with relatively high (77–78 %) sequence identity. Alignment of these sequences with *rcc2* and *rcg3* gene-specific primers (Online Resource 1) showed very low or no sequence similarity of these sequences at the 3′ end of the primers used to amplify the rice chitinase genes. These in silico findings were confirmed experimentally as none of the untransformed control plants yielded any amplification products with the *rcc2* or *rcg3* gene-specific primers. Furthermore, the optimized Southern hybridization procedure using a probe that spans part of the promoter region and applying more stringent washing conditions (see Sect. Materials and methods: Southern blot hybridization) successfully eliminated background signals caused by banana chitinase sequences. Southern hybridization revealed that the number of transgene integrations varied from one to five per line and, as evidenced by the hybridization pattern, each transgenic line represented a unique transformation event. In summary, this is the first report of successful stable transformation of ‘Gros Michel’ or even any non-Cavendish dessert banana (AAA genomic group).

No differences in growth and morphological characteristics were observed between transgenic and non-transgenic plants, therefore the 17 transgenic lines selected were tested by the in vitro leaf disk bioassay (LDA). During the last decade, these bioassays became a useful tool for screening transgenic plants for disease resistance (Kumar et al. 2003; Marchant et al. 1998; Takatsu et al. 1999). Two main categories of LDA can be distinguished, detached (Abadie et al. 2009; Agnola et al. 2003; Bussey and Stevenson 1991; Cohen 1993) and attached LDAs (Donzelli and Churchill 2007; Meeley et al. 1992; Twizeyimana et al. 2007a, b). In case of banana, rapid mass screening with attached leaves is difficult to carry out because of requirements in space and screenhouse conditions (e.g., high humidity and temperature) for efficient control of inoculations. Since banana, in addition, has a long generation time (up to 2 years in greenhouse) and large plant size (height up to 10 m with an enormous leaf area), a reliable space- and time-saving pre-screening method of transgenic lines for resistance would facilitate the identification of promising lines before further testing in the field. Therefore, we chose detached leaves in this study. The first visible symptoms of BLSD under normal field conditions appear 2–4 weeks after infection and the entire disease cycle spans 1–3 months (Foure et al. 1990). Although ascospore production does not occur in this test, one of the most important parameters of an in vitro LDA for the banana/*M. fijiensis* pathosystem is the ability to keep the leaf disks healthy for at least 2–3 months because it allows to monitor a slower increase of the infected leaf area, i.e., partial resistance. Previously described banana LDAs where benzimidazole (BIM) was applied as a retardant of senescence as well as a fungicide to prevent undesirable fungal infection lasted 70 dpi (Abadie et al. 2008, 2009). However, in our hands BIM was unable to prevent early (prior to 70 dpi) necrosis of the leaf disks (data not shown). By replacing BIM with GA_3_, with no negative effect on the disease development, we managed here to preserve intact banana leaf disks for more than 100 days. Twizeyimana et al. (2007a) concluded that a GA_3_ concentration of 5 mg L^−1^ is adequate to significantly reduce the chlorosis on banana leaf disks, but they observed only 52 days of incubation. Similar to a greenhouse test where successful disease development can take longer incubation period [2–3 months (Churchill 2010; Donzelli and Churchill 2007)], we have found that an 8 mg L^−1^ GA_3_ supplement provides sufficient longevity for leaf disks to fulfill this condition without deteriorating. Although there are suggestions in other plant/fungus interactions (Takahashi et al. 2005; Yamamoto et al. 2000) that transgene expression just postpones symptom development and prolonging the incubation time results in similarly severe symptoms in transgenic plants as in non-transgenic controls, we observed this phenomenon only in the most susceptible transgenic line (GM.RCG3.09) during our LDA (up to 108 dpi). This result strengthens our conclusion that the rice chitinase transgene is able to provide resistance against BLSD in banana under controlled conditions.

In previous reports, over-expression of the rice *rcc2* chitinase gene resulted in various levels of resistance among transgenic lines challenged with fungal pathogens (Kishimoto et al. 2002; Takatsu et al. 1999). Similarly, in our study the tested transgenic banana lines exerted a wide range of resistance against *M. fijiensis*, but an unambiguous delay in the lesion development was observed in all lines except GM.RCG3.09. Under controlled conditions the growth of *M. fijiensis* is thus evidently inhibited in leaf disks of transgenic banana plants carrying a rice chitinase gene. The field resistance of ‘Calcutta 4’ against BLSD was recapitulated in the LDA with a negligible relative infected leaf area well below 1 % at 108 dpi. Using an optimized in vitro LDA, nine transgenic lines with a high level of resistance were identified. The necrotic leaf area was reduced with 73–94 % in these transgenic lines at 108 dpi compared to that of the susceptible control. The fact that all of these lines carry the *rcg3* gene of which the product is known to be targeted extracellularly is striking. In contrast, Nishizawa et al. (1999) did not find a notable difference between transgenic rice plants expressing one of the two rice chitinases (RCC2 or RCG3) when they were challenged with rice blast (*Magnaporthe grisea*), another hemibiotrophic fungus. Nevertheless, a conclusion on the effect of the subcellular targeting of the two chitinases on the acquired resistance level would be premature since we had only two *rcc2* lines available for investigation. Studies on compatible interactions demonstrate that *M. fijiensis* enters susceptible banana leaves through the stomata, and during a long initial biotrophic phase the fungus colonizes the intercellular spaces between mesophyll cells prior to the formation of haustoria (Cavalcante et al. 2011). We hypothesize that transgenic banana plants secreting chitinase at an elevated level to the extracellular space (RCG3) might be reacting faster to pathogen penetration than those expressing the vacuole-type chitinase (RCC2). This early attack of the fungal cell wall by the chitinase can lead to early release of *N*-acetyl-d-glucosamine oligomers, which are assumed to function as elicitors to activate defense-related response of surrounding cells (Kumar et al. 2003; Nishizawa et al. 1999).

Expression analysis of the *rcg3* chitinase gene by Western blots was conducted on eight resistant lines. Since the antibodies were raised against a 291 amino acid (AA) sequence of the RCC2 protein [340 AA in total; (Nishizawa et al. 1999)] the protein sequence similarity of 68 % between this RCC2 sequence and the complete RCG3 protein (320 AA) was apparently sufficient to cross-react with the highly homologous RCG3 chitinase protein. This was empirically confirmed by additional Western blot experiments. Purified RCC2 and RCG3 protein samples were run in parallel on two identical gels and each was hybridized with RCC2 antibody. A specific 32 kDa band was detected in each protein-antibody combination demonstrating full cross-reactivity (data not shown).

A distinctive signal of the correct size was detected in two transgenic lines and was absent in the untransformed control. In line GM.RCG3.32, which exhibited the highest resistance in the LDA, the RCG3 chitinase level increased during the incubation period. In contrast, a decrease in RCG3 was detected in the other Western-positive line, GM.RCG3.01, which was the least responsive in the LDA among the resistant lines. Hence, a clear relation was observed in these two lines between chitinase expression level and the resistance assessed over time by LDA. Rice chitinase expression was detected at all four time points investigated, which demonstrates the stability of over-expression at least for 78 days. Chitinase expression in the remaining six transgenic lines was below the detection limit despite their resistance as earlier detected in LDA. Indeed, Western analysis was done on plants multiplied from shoot tip cultures maintained in vitro by six to eight additional subculture steps (in total, approximately 3 years) compared to the plants used for the LDA. It is therefore conceivable that the loss of transgene expression in these lines was due to the long propagation time. In vitro culture has long been known to induce unstable expression (Bettany et al. 1998; Meng et al. 2006) or deletion of transgenes (Risseeuw et al. 1997), including banana as well (Matsumoto et al. 2007). In ongoing experiments in our laboratory we are investigating the probability of this scenario prior to further development and testing under field conditions of promising transgenic banana lines.

In conclusion, we produced proof-of-concept transgenic ‘Gros Michel’ banana plants and demonstrated for the first time that overexpression of a plant chitinase alone can effectively enhance resistance in banana against *M. fijiensis.* Our results also confirmed that GA_3_ is a superior supplement for incubation media in a bioassay as it prolonged the life span of banana leaf disks up to 108 days. Finally, the optimized in vitro leaf disk bioassay is an effective tool for early selection of transgenic banana lines for disease resistance.

## Electronic supplementary material

Below is the link to the electronic supplementary material.

Supplementary material 1 (DOCX 12 kb)

Supplementary material 2 (DOCX 36 kb)
