# Supplementary material for: Expression of a rice chitinase gene in transgenic banana (‘Gros Michel’, AAA genome group) confers resistance to black leaf streak disease
Source: Transgenic Res. 2012 Jul 13;22(1):117–30. doi: 10.1007/s11248-012-9631-1 (PMC3525978; doi:10.1007/s11248-012-9631-1)
Supplement: Supplementary file 1 — Supplementary material 1 (DOCX 12 kb) [file 11248_2012_9631_MOESM1_ESM.docx]

**Online Resource 1**

**Title:** Expression of a Rice Chitinase Gene in Transgenic Banana (‘Gros Michel’, AAA genome group) Confers Resistance to Black Leaf Streak Disease

**Journal:** Transgenic Research

**Authors:** Gabriella Kovács, László Sági, Géraldine Jacon, Geofrey Arinaitwe, Jean-Pierre Busogoro, Els Thiry, Hannelore Strosse, Rony Swennen, Serge Remy

**Corresponding author:** [Serge.Remy@biw.kuleuven.be](mailto:Serge.Remy@biw.kuleuven.be); Laboratory of Tropical Crop Improvement, Department of Biosystems, Faculty of Bioscience Engineering, Katholieke Universiteit Leuven, Kasteelpark Arenberg 13, bus 2455, 3001 Leuven, Belgium

**Supplementary Table**

**Table S1.** List of transgene-specific primer pair sequences

| **Transgene** | **Primer pair** |
| --- | --- |
| *rcc2* | 5´-GCG GGT TCT ACA CCT ACG AG-3´  5´-GCG TCA TCC AGA ACC ACA-3 |
| *rcg3* | 5´-CCG CTA AGG GCT TCT ACA CC-3´  5´-GCG TCA TCC AGA ACC AGA AC-3 |
| *hpt* | 5´-ACT TCT ACA CAG CCA TCG GTC-3´  5´-GAC CTG CCT GAA ACC GAA CTG-3 |
