# Supplementary material for: Expression of a rice chitinase gene in transgenic banana (‘Gros Michel’, AAA genome group) confers resistance to black leaf streak disease
Source: Transgenic Res. 2012 Jul 13;22(1):117–30. doi: 10.1007/s11248-012-9631-1 (PMC3525978; doi:10.1007/s11248-012-9631-1)
Supplement: Supplementary file 2 — Supplementary material 2 (DOCX 36 kb) [file 11248_2012_9631_MOESM2_ESM.docx]

**Online resource 2**

**Title:** Expression of a Rice Chitinase Gene in Transgenic Banana (‘Gros Michel’, AAA genome group) Confers Resistance to Black Leaf Streak Disease

**Journal:** Transgenic Research

**Authors:** Gabriella Kovács, László Sági, Géraldine Jacon, Geofrey Arinaitwe, Jean-Pierre Busogoro, Els Thiry, Hannelore Strosse, Rony Swennen, Serge Remy

**Corresponding author:** [Serge.Remy@biw.kuleuven.be](mailto:Serge.Remy@biw.kuleuven.be); Laboratory of Tropical Crop Improvement, Department of Biosystems, Faculty of Bioscience Engineering, Katholieke Universiteit Leuven, Kasteelpark Arenberg 13, bus 2455, 3001 Leuven, Belgium


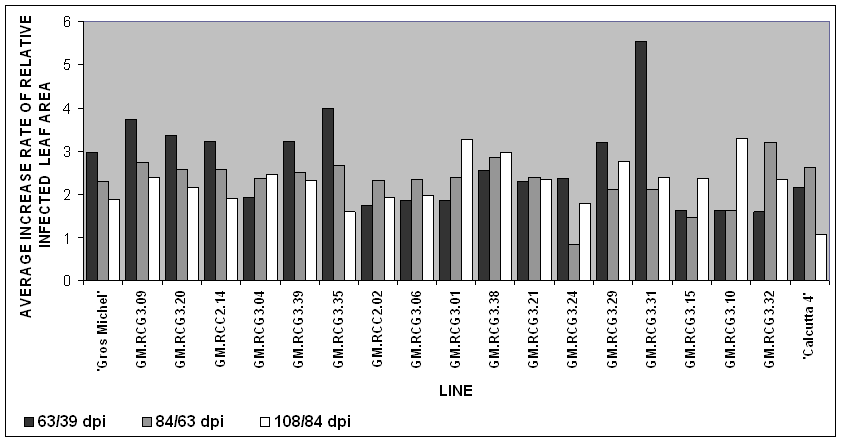


**Supplementary Fig. S1** Average increase rate of relative infected leaf area of leaf disks over three consecutive time periods.

Experimental details under Fig. 2 and the order of lines is identical to that in Fig. 3. For each time period (39-53 dpi, 53-84 dpi and 84-108 dpi) the average relative infected leaf area at the end of the time period was divided by that at the start of the time period to obtain the average increase rate of relative infected leaf area. RCG3, transgenic lines with rice chitinase *rcg3* and RCC2, transgenic lines with rice chitinase *rcc2;* dpi, days post inoculation
